# MicroRNA profile of paclitaxel-resistant serous ovarian carcinoma based on formalin-fixed paraffin-embedded samples

**DOI:** 10.1186/1471-2407-13-216

**Published:** 2013-04-30

**Authors:** Xiao Li, Yaer Lu, Yaxia Chen, Weiguo Lu, Xing Xie

**Affiliations:** 1 Women’s Reproductive Health Laboratory of Zhejiang Province, Women’s Hospital, School of Medicine, Zhejiang University, Hangzhou, China; 2 Department of Gynecologic Oncology, Women’s Hospital, School of Medicine, Zhejiang University, No.1 Xueshi Road, Hangzhou, Zhejiang, 310006, China; 3 Zhejiang Provincial People’s Hospital, Hangzhou, Zhejiang, China

**Keywords:** Ovarian carcinoma, Chemoresistance, Formalin-fixed paraffin-embedded (FFPE), microRNA

## Abstract

**Background:**

To assess the feasibility of validating microRNA (miRNA) profile related to paclitaxel-sensitivity in formalin-fixed paraffin-embedded (FFPE) samples of serous ovarian carcinoma (OC) patients.

**Methods:**

Deregulated miRNAs identified by miRNA microarray were further detected in 45 FFPE OC samples using Realtime PCR. Correlations between paired FFPE and frozen tumor samples were analyzed. Survival times were compared between 6 high and low miRNAs groups. Western blot and luciferase reporter assay were used for validating the target of miRNA.

**Results:**

Sixteen up-regulated miRNAs and twenty-three down-regulated miRNAs were revealed in pacilitaxel-resistant ST30 cells. The up-regulated miRNAs (miR-320a, 22 and 129-5p) and down-regulated miRNAs (miR-9, 155 and 640) were confirmed in paclitaxel-resistant FFPE tumor samples, compared with paclitaxel-sensitive samples. Higher miR-9 and miR-640 showed better survival time in OC patients. Expressions of miR-9, 155 and 22 in FFPE samples were closely mimicked by those in frozen tissues. RAB34 was validated as a direct target of miR-9.

**Conclusions:**

We validated miRNA profile in pacilitaxel-resistant OC using FFPE samples, which might enable treatment stratification and help us to predict outcomes in OC patients. FFPE samples are feasible materials for miRNA research.

## Background

Ovarian carcinoma (OC) is the leading cause of death among patients with gynecologic cancer. Despite initial responsiveness to the chemotherapeutic drugs, most patients eventually develop chemoresistance and succumb to their diseases [1,2]. Drug resistance remains a formidable problem in managing cancer patients. It is clinically important to identify biomarkers that could assist in predicting which patients will respond to chemotherapy, and, which patients will remain refractory to standard treatment.

microRNA (miRNA)s are small noncoding RNAs involved in the initiation and progression of human cancer [3]. As modulators of protein expression they may operate as oncogenes and tumor suppressors [4]. miRNA expression profiles are useful for assessment of the prognosis and chemosensitivity of human cancer. For example, when cisplatin resistance was specifically examined for miRNA expression, 34 miRNAs with statistically significant changes were found in tumors from responders compared with those of non-responders [5]. Among them, Let-7i was the most down-regulated miRNA in the chemo-resistant patients. In another study, elevation of miR-214 was found to be responsible for development of resistance to cisplatin. Blocking miR-214 expression enhanced the sensitivity of A2780 cells to cisplatin-induced apoptosis [6]. Eitan et al. also found an array of tumor specific miRNAs associated with response to platinum [7]. However, few studies have focused on tumor miRNA expression patterns associated with paclitaxel-resistance. Several miRNAs are differentially expressed in paclitaxel-resistant and sensitive OC cell lines, when we detected the miRNAs expressions using miRNA microarray in present study. This raises the question that whether a miRNA profile representing paclitaxel-resistance could be useful for prognosticating clinical chemo-resistance in OC patients.

As we know, routine histology processing uses formalin fixation to preserve clinical specimens, which provides a rich resource of tissues linked to clinical databases. In addition, any biomarker developed from formalin-fixed paraffin embedded (FFPE) samples could be more readily translated into clinical practice than frozen tissues. However, most studies of miRNAs still use frozen tissues, despite prospective collection of frozen samples is limited and inefficient [8,9]. The most important reason is that RNA is degraded in tissues before, during, and after formalin fixation [10,11]. Interestingly, miRNAs appear to be better preserved, perhaps because of their shorter lengths. Detecting miRNAs using FFPE tissues has been successful for colon, breast and ovarian cancers [12-14]. In present study, we try to validate a panel of miRNAs associated with pacilitaxel-resistant OC using FFPE samples, and to determine that whether certain miRNAs might predict response to chemotherapy and prognosis. Furthermore, to show the suitability of routine FFPE tissue for comprehensive miRNA expression analyses using Realtime PCR, we also compared expressions of miR-9, 155 and 22 in FFPE samples with those in paired frozen samples from the same patients.

## Methods

### Patient’s characteristics

FFPE tumor samples (N=45) were collected in our hospital between Feb 2001 and Mar 2009, from the patients surgically treated for primary serous papillary ovarian adenocarcinoma and received chemotherapy including paclitaxel after primary surgery. Matched frozen samples (N=10) from the same patients were also obtained (immediately snap-frozen in liquid nitrogen and stored at −80°C). Only tumor samples with a minimum of 70% tumor tissue content were included. All pathology slides were evaluated by an expert pathologist. Patients’ characteristics are listed in Table 1. Patients with progressive disease during primary chemotherapy or those who suffered recurrent disease within 6 months of completing primary chemotherapy were termed paclitaxel-resistant. Patients with recurrence beyond 6 months or without recurrence were termed paclitaxel-sensitive. Overall survival time (OS) was calculated as the time from surgery to the last follow-up date or death. Progression free survival time (PFS) was calculated as the time from surgery to the time of detected recurrence or progression. The study was approved by the ethical committee of women’s hospital, school of medicine, Zhejiang University (Reference number 20070430).

**Table 1:** Clinical characteristics of 45 ovarian carcinoma patients

	**Sensitive**	**Resistant**
Cases number (N)	24	21
Age (range, years)	52(41–67)	52(32–68)
FIGO Stage		
I(N)	3	0
II(N)	1	2
III(N)	18	17
IV(N)	2	2
Tumor Grade		
1(N)	1	1
2(N)	7	2
3(N)	16	18
Recurrence		
No (N)	2	0
Yes (N)	22	21
PFS (Range,months)	22(12–78)	6(3–11)
OS (Range, months)	42.5(18–144)	17(7–65)
Survival		
No (N)	12	20
Yes (N)	12	1

### Cell culture and transfection

Paclitaxel-resistant cell line SKOV3-TR30 (ST30) was induced from SKOV3 (American Type Culture Collection, Manassas, VA, USA). Both were maintained in McCoy’s 5A medium supplemented with 10% fetal bovine serum. For ST30 cells, 30nm paclitaxel was withdrawn at 2 weeks before experiment. For regulation of miR-9, ST30 cells were transfected with 50nM miR-9 mimic or its negative Control (Ribobio, GuangZhou, China) by using Lipofectamine 2000 (Invitrogen, Carlsbad, CA, USA) in accordance with the manufacturer’s instructions.

### RNA extraction and microRNA microarray

Total RNA was extracted from 45 FFPE tumor samples and 10 FFPE matched frozen tumor samples from the same patients. For snap-frozen tissues, total RNA was isolated by TRIzol(Invitrogen, California, USA). For FFPE tissues, total RNA was extracted by RecoverAll™ Total Nucleic Acid Isolation Kit (Ambion, Austin, TX, USA), according to the manufacturer’s instructions. Briefly, eight 10-um-thick sections of FFPE samples were deparaffinized with xylene and washed in ethanol. The tissues were digested with protease and treated with DNase. After washing, total RNA was eluted with distilled water. The quantity and quality of RNA were measured by Nanodrop 2000 thermo scientific spectrophotometer (NanoDrop Technologies, Wilmington, DE, USA).

miRNA expression profiling of SKOV3 and ST-30 cells were detected using the miRCURY LNA Array (version 11.0) system. RNA samples were labeled with the Exiqon miRCURY™ Hy3™/Hy5™ Power labeling kit and hybridized on the miRCURY LNA Array (version 11.0) station. Scanning was performed with the Axon GenePix 4000B microarray scanner. GenePix pro version 6.0 was used to read image raw intensity of the image. The ratio of red signal to green signal was calculated after background subtraction and normalization using the global Lowess (Locally Weighted Scatter plot Smoothing) regression algorithm (MIDAS, TIGR Microarray Data Analysis System). Between slides normalization was performed by scale normalization to reduce between-slide variability. The experiments were performed in triplicate and repeated three times. The statistical significance of differentially expressed miRNAs was analyzed by fold change and *t*-test. The threshold value we used to screen differentially expressed miRNAs was a fold change>1.5 or <0.67.

### Realtime RT-PCR validation

The most up-regulated and down-regulated miRNAs identified by microarray were further validated in 45 FFPE tumor samples using realtime RT-PCR. To corroborate the validity of miRNAs expression of FFPE samples, three miRNAs were detected in 10 matched FFPE and snap-frozen samples from the same patients. Correlations of miRNAs expressions between matched FFPE and frozen tumor samples were analyzed.

RT reactions and realtime PCR were performed according to the manufacturer’s protocols (TaKaRa, DaLian, China). All RT reactions, including no-template controls were run in a PTC-200 Peltier Thermal Cycler (MJ Research, Waltham, MA, USA). miRNA levels were quantified with the ABI Prism 7900HT sequence detection system (Applied Biosystems, Fostercity, CA, USA). U6 snRNA served as endogenous control. The primers for miRNAs and U6 were obtained from Ribobio (Ribobio, GuangZhou, China). Comparative realtime PCR was performed in triplicate, including no-template controls. Relative expression was calculated using the 2-ΔΔct method.

### Western blotting

At 72 h after transfection, total protein extracts from the cells were prepared. Total protein was separated by SDS–PAGE and transferred electrophoretically to a nitrocellulose membrane. The membrane was blocked with 5% nonfat dried milk in TBST buffer, and then incubated overnight at 4°C with primary antibodies against RAB34 (SC-27442, 1:250, Santa Cruz) and GAPDH (sc-25778, 1:1000, Santa Cruz) respectively. Blots were washed with TBST buffer and incubated with secondary antibody. The protein bands were visualized using the EZ-ECL kit (Biological Industries, Kibbutz Beit-Haemek, Israel). GAPDH was used as an endogenous control. The experiments were repeated three times.

### Dual luciferase reporter assay

The 3′-untranslated region (UTR) of RAB34 (379BP) mRNA containing the miR-9 binding site were PCR amplified, and then cloned into the pmiR-RB-REPORT™ dual luciferase reporter vector (Promega) at the 3′ end of the coding sequence of renilla luciferase using XhoI and NotI restriction sites. To produce the mutations of the miR-9 targeting site, site-directed Gene Mutagenesis Kit (Beyotime, Jiangsu, China) was used according to the manufacturer’s protocol. All inserted or mutated sequences were confirmed by sequencing. The primers and mutation primers are listed in Table 2. All primers were synthesized by Ribobio. Cotransfection of miRNA mimic or its negative control (50 nM) and different reporter vectors (50nM) to 293T cells was performed using Lipofectamine 2000. The luciferase activities were measured at 48 hours after transfection using VICTOR3™ multilabel readers (Perkinelmer, Waltham, MA, USA), and the renilla luciferase activities were normalized to firefly luciferase activities. The experiments were performed in triplicate and repeated three times.

**Table 2:** The sequences of primers in the dual luciferase reporter assay

**Primer**	**Sequence 5′-3′**
RAB34-WT-F	CCG CTCGAGGGGCTGAGGAGACTGTTC
RAB34-WT-R	GAATGCGGCCGCGCTCGTAACAAAGAAATTTTAATGCATAAG
RAB34-Mut-F	TTATCCAG**TCGATAC**TGCTGCCTCTTGGTGGCAGTA
RAB34-Mut-R	CAGCAGTATCGACTGGATAAAGTCAGTGCAAATGT

*WT*: wild type; *F*: forward, *R*: reverse; *Mut*: mutation. The mutation site in the Mut-primer is bold.

### Statistical analysis

Student’s *t*-test was used to assess miRNAs expression in FFPE samples and dual luciferase reporter assay. Kaplan-Meier survival functions and log-rank test were used to assess progress-free survival and overall survival time based on mean expression levels of 6 miRNAs. Pearson’s correlation was calculated to compare the PCR results of FFPE samples with those of paired frozen samples. All statistical analyses were two-sided and performed with SPSS 11.5 software package. P-values less than 0.05 were considered statistically significant.

## Results

### Confirmed paclitaxel-resistant miRNA profile using FFPE samples

**Figure 1:**
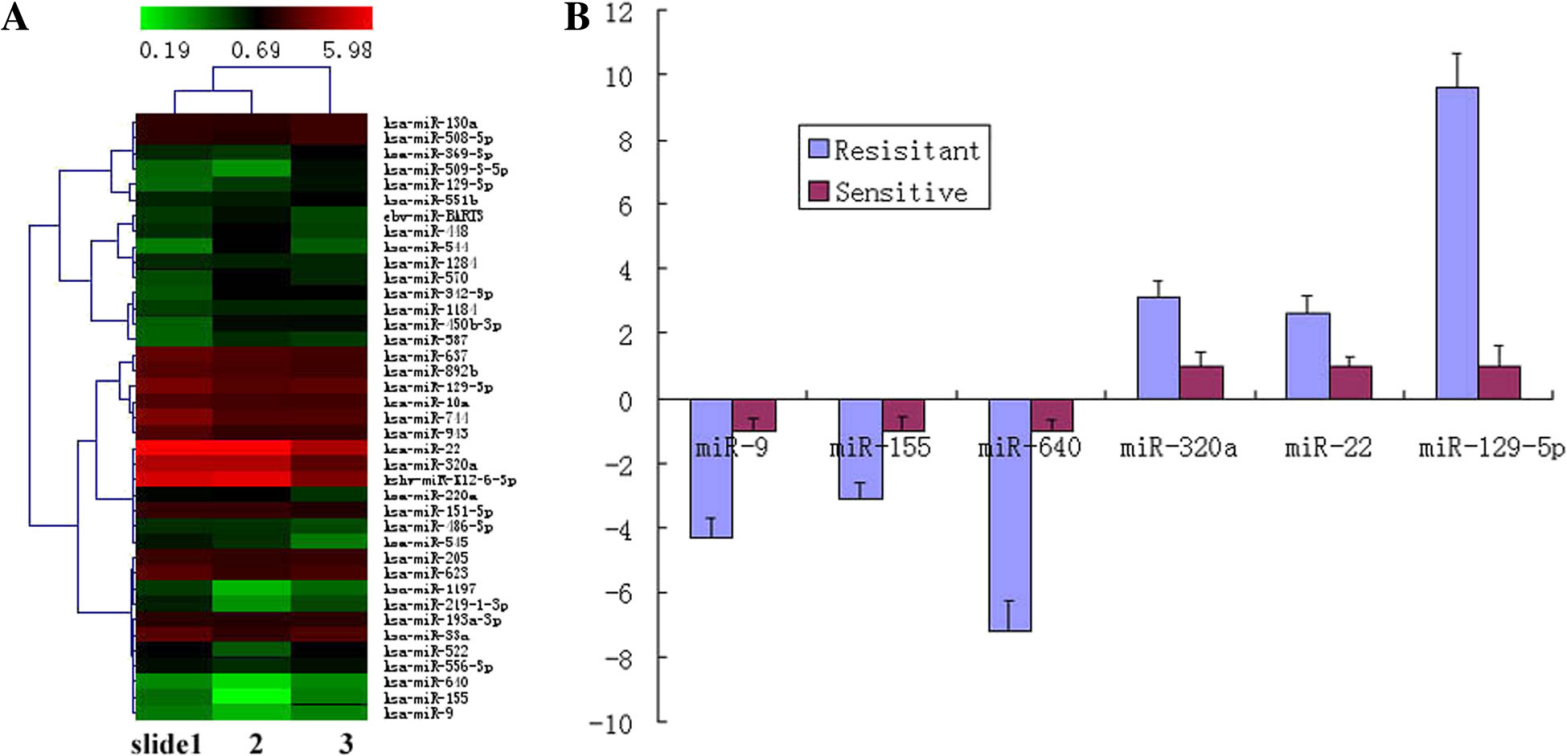
**The miRNA profile associated with paclitaxel-resistance and survival of OC patients. A**, The heat map of miRNA microarray expression of SKOV3 and ST30 cells. The heat map diagram shows all the deregulated miRNAs passed *T* test. Each row represents a miRNA and each column represents a pair of samples (n=3). The color scale showmn at the top illustrates the relative expression level of a miRNA in the certain slide: red color represents a higher expression level than control sample; green color represents a lower expression level than the control sample. **B**, Realtime RT-PCR for miR-9, 155, 640 miR-320a, 22 and 129-5p in 45 serous ovarian adenocarcinoma FFPE tissues. The error bars represent SEM. The miRNAs are significantly deregulated in paclitaxel-resistant tissues compared with paclitaxel-sensitve tissues. (*P*=0.009, 0.027, 0.001, 0.019, 0.025 and 0.007 respectively).

The miRNA microarray revealed that 39 miRNAs showed more than 1.5 fold deregulation between the pacilitaxel resistant and sensitive cell lines (Figure 1A). Among them, miR-320a, 22 and 129-5p are the most up-regulated miRNAs, and miR-9, 155 and 640 are the leading down-regulated miRNAs.

**Figure 2:**
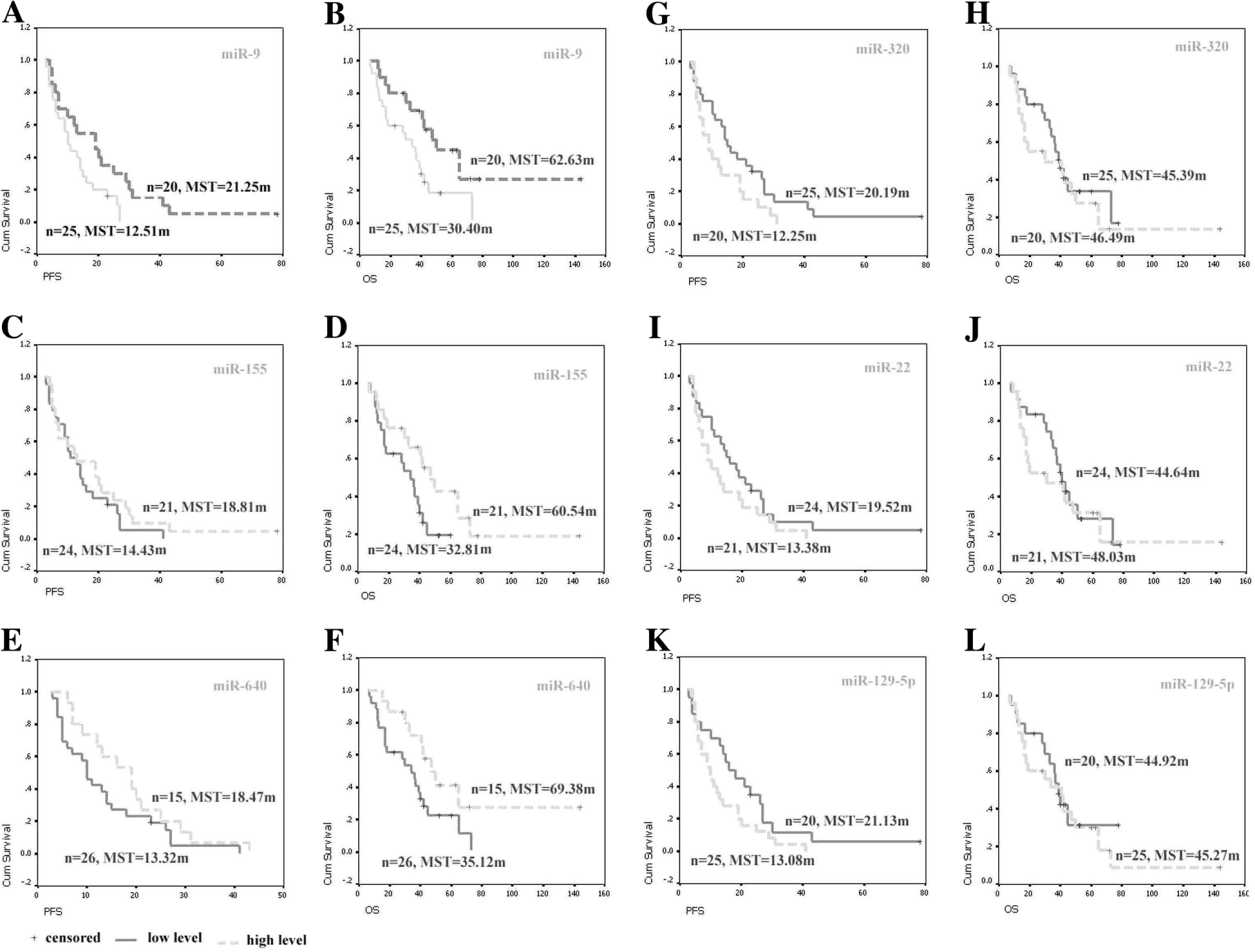
**PFS and OS of 45 OC patients by the level of 6 deregulated miRNAs. A**-**B**, The PFS and OS in patients with higher miR-9 level are significantly longer than lower miR-9, *P*=0.0267 and 0.021. **C**-**D**, The PFS and OS in patients with higher miR-155 level are longer than lower miR-155, but not significantly, *P*=0.289 and 0.060. **E**, The PFS in patients with higher miR-640 level are longer than lower miR-640, but not significantly, *P*=0.148. **F**, The OS in patients with higher miR-640 level are significantly longer than lower miR-9, *P*=0.043. G-L, The PFS in patients with higher miR-320a, 22 and 129-5p level are shorter than lower miRNAs, but not significantly, *P*=0.055, 0.175 and 0.717. The OS in patients with higher miR-320a, 22 and 129-5p are no difference compared with lower miR-320a, 22 and 129-5p, *P*= 0.465, 0.550 and 0.646 in turns. MST, mean survival time in months.

To determine the significance of these miRNAs, realtime PCR was performed using 45 FFPE ovarian carcinoma samples. miR-320a, 22 and 129-5p were significantly up-regulated in FFPE paclitaxel-resistant tumor samples (*P*=0.019, 0.025, 0.007), while miR-9, 155 and 640 were significantly down-regulated (*P*=0.009, 0.027 and 0.001 respectively) (Figure 1B), compared with paclitaxel-sensitive samples. Furthermore, we divided all tissues into high and low miRNAs expression groups based on the mean values of 6 miRNAs, and found longer PFS (21.25 months, *P*=0.027) and OS (62.63 months, *P*=0.021) in patients with high miR-9 expression than those with low miR-9 level (12.51 months and 30.40 months, respectively) (Figure 2A and B). Higher miR-640 showed significantly longer OS (69.38 months compared with 35.12 months, *P*=0.043, Figure 2F). Higher miR-155 and 640, lower 320a, 22 and 129-5p showed higher PFS while higher-155 showed longer OS, although not significantly (Figure 2C-E, G, I and K). This data validates a miRNA profile that is characteristically expressed in paclitaxel-resistant OC tissues and suggests poorer prognosis for OC patients.

### Consistent miRNA expressions validated between paired frozen and FFPE samples

To further corroborate the validity of miRNA profile of FFPE samples, realtime RT-PCR were repeated using pairs of frozen and FFPE tissues from the same patients. Correlation analysis of miR-9, 155 and 22 levels in FFPE and frozen samples from the same patients showed significant correlationship of the paired samples, with Pearson’s rho of 0.88, 0.89 and 0.87 respectively (all *P*= 0.001). Thus, miRNA expression studies can be reliably performed with routinely obtained pathological materials and the results are similar to the yield from snap-frozen tissues.

### RAB34 is one of the targets directly regulated by of miR-9

**Figure 3:**
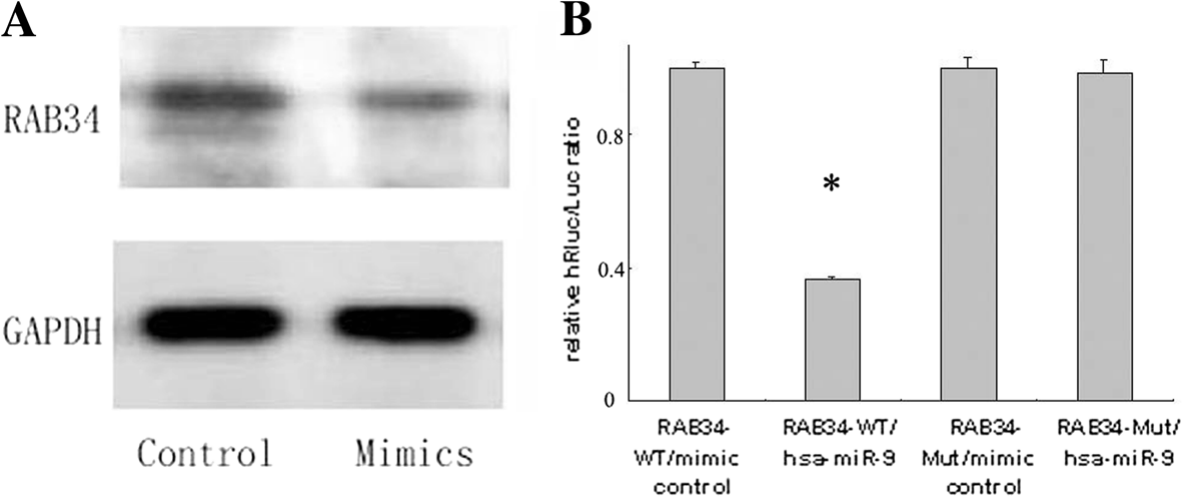
**RAB34 is the direct target of miR-9. A**, Western blot analysis of RAB34 in ST30 cells transfected with miR-9 mimic or negative control. GAPDH was used as house-keeping gene. **B**, Dual luciferase reporter assay. 293T cells were transfected with RAB34-wild type 3′UTR vectors or mutant 3′UTR vectors together with miR-9 mimic or its negative control. Luciferase activity was measured 48 h after co-transfection, and the error bars represent SD. A decrease of the luciferase activity was observed in miR-9 over-expressing cells (* *P*= 0.000).

The relationship between miRNAs level and prognosis of OC patients triggered us to investigate the biological consequence of altered miR-9. We predicted some gene targets of miR-9 involved with important cellular signal pathways using TargetScan database (http://www.targetscan.org, Released 6.0, Nov 2011). RAB34 contained the putative miR-9 binding site, indicating that it might be one of the targets regulated by miR-9. Western blot analysis showed that overexpression of miR-9 by specific mimic, down-regulated the expression of RAB34 in ST30 cells (Figure 3A). Using dual luciferase reporter assay, we further found that the relative luciferase activities were significantly reduced in cells transfected with RAB34 WT-3′UTR/mirR-9 mimic vectors compared with those transfected with RAB34 WT-3′UTR/mirR-9 mimic control (*P*=0.000), but the relative luciferase activities were not altered in cells transfected with RAB34 Mut-3′UTR/miR-9 mimic or RAB34 Mut-3′UTR/miR-9 mimic control (Figure 3B). Collectively, these results validate that miR-9 binds directly to the sites within the 3′UTR of RAB34 and RAB34 is the direct target of miR-9.

## Discussion

Despite multiple new approaches to treatment, the high death rates from OC remain largely unchanged with a 5-year overall survival of only 30–39% [15]. Dramatically poor prognosis of OC patients is due to the development of chemo-resistance. As shown in Table 1, the median PFS in paclitaxel-sensitive group is 22 months, significantly longer than that in paclitaxel-resistant group (6 months, *P*=0.000), and the median OS in paclitaxel-sensitive group is also significantly longer than that in paclitaxel-resistant group (42.5 vs 17 months, *P*=0.000). Therefore, identifying patients who can benefit from the chemotherapy will have significant clinical implications.

miRNA represents a new class of small non-coding RNA that regulates multiple gene expression. During tumorigenesis and tumor progression, up-regulated miRNA could potentially target and down-regulate tumor suppressor genes, whereas down-regulated miRNA could potentially increase the expression of oncogenes [16]. Increasing evidence supports that miRNA may influence the response of ovarian cancer to chemotherapy [17-19]. However, all these evaluations used frozen tissues. In present study, we provide the first characterization of the paclitaxel-resistant miRNA profiling of 45 FFPE samples from primary serous papillary ovarian adenocarcinoma patients using realtime PCR assay. Three top deregulated miRNAs were selected for validation using 10 paired FFPE and frozen samples from the same patients. Importantly, the changes predicted for these 3 miRNAs using the paired samples from the same patients were significantly correlated. Thus, these data demonstrate that FFPE samples may be used as a feasible material for miRNA expression research. Although different types of tumors may have the same miRNAs markers, there are specific miRNAs in tumors from different cellular origins [20]. In present study, the majority of the differentially expressive miRNAs have not been reported before, such as miR-9 and miR-640. This discrepancy may be attributed to different patient population and inter-experimental variations. Our study focused on primary serous papillary ovarian adenocarcinoma patients and paclitaxel-sensitivity, whereas previous studies were involved different histological types of ovarian cancer and chemotherapy agents [5-7].

Specific data may assist in tailoring treatment to each patient’s specific clinical situation during the initial management of their disease and also offer the opportunity for better counseling regarding prognosis. Based on the confirmed top deregulated miRNAs in paclitaxel-resistant ovarian carcinoma, we further analyzed the associations of different miRNAs expression and survival outcomes after stratifying the OC patients into high and low miRNAs’ levels. Our analyses showed that patients those with high miR-9 and 640 level survived better than those with low results. Moreover, deregulation of miR-155, 320a, 22 and 129-5p were found to be associated with shorter PFS of the patients, although not significantly. Thus, our findings suggest that a miRNA profile may help us to distinguish patients who will respond to paclitaxel treatment and provide biomarker of poor prognosis in OC patients. However, given the small number of patients involved in the study, these results need to be interpreted with caution.

Understanding the regulatory role of miRNA may lead to better understanding of the molecular events involved in different biological processes, and,lead to the development of diagnostic tools and a novel class of drug targets for therapeutic interventions. According to the TargetScan database, there are over 1200 predicted hsa-miR-9 targets, including some familiar oncogenes. Among them, RAB34 has been confirmed as a direct target of miR-9 in OC using western blot and dual luciferase reporter assay. As a member of RAS oncogene family, RAB34 is a guanosine triphosphatase (GTPases) that can regulate budding junction and fusion of vesicle in exocytosis and endocytosis pathway [21]. Luo et al. [22] has found that miR-9 was down-regulated and can be the modulator of RAB34 in gastric carcinoma, which was concordant with our finding.

## Conclusions

There is still work to be done for better defining the mechanisms of miRNA-associated paclitaxel resistance and its potential as a future target for therapy in OC. However, our results support the conclusions that miRNA profile allows meaningful subclassification of paclitaxel-resistant ovarian tumors and prognosis prediction. FFPE samples are convenient and feasible materials for miRNA study. In addition, we find miR-9 might be correlated with the paclitaxel-sensitivity through targeting RAB34. Hopefully, these findings can ultimately be used to improve ovarian cancer patient outcome.

## Competing interests

The authors declare that they have no competing interests.

## Authors’ contributions

XL carried out cell culture, PCR, Western Blot and drafted the manuscript. YL and YC collected the patient’s materials and followed up. WL carried out Dual luciferase reporter assay. XX conceived the study and revised the manuscript. All authors read and approved the final manuscript.

## Pre-publication history

The pre-publication history for this paper can be accessed here:

http://www.biomedcentral.com/1471-2407/13/216/prepub
